# The Crystal Structure of *Arabidopsis* VSP1 Reveals the Plant Class C-Like Phosphatase Structure of the DDDD Superfamily of Phosphohydrolases

**DOI:** 10.1371/journal.pone.0049421

**Published:** 2012-11-14

**Authors:** Yuhong Chen, Jia Wei, Mingzhu Wang, Zhubing Shi, Weimin Gong, Min Zhang

**Affiliations:** 1 School of Life Sciences, Anhui University, Hefei, Anhui, China; 2 Center for Protein Sciences, Institute of Biophysics, Chinese Academy of Sciences, Beijing, China; 3 Institute of Biochemistry and Cell Biology, Shanghai Institutes for Biological Sciences, Chinese Academy of Sciences, Shanghai, China; University of South Florida College of Medicine, United States of America

## Abstract

*Arabidopsis thaliana* vegetative storage proteins, VSP1 and VSP2, are acid phosphatases and belong to the haloacid dehalogenase (HAD) superfamily. In addition to their potential nutrient storage function, they were thought to be involved in plant defense and flower development. To gain insights into the architecture of the protein and obtain clues about its function, we have tested their substrate specificity and solved the structure of VSP1. The acid phosphatase activities of these two enzymes require divalent metal such as magnesium ion. Conversely, the activity of these two enzymes is inhibited by vanadate and molybdate, but is resistant to inorganic phosphate. Both VSP1 and VSP2 did not exhibit remarkable activities to any physiological substrates tested. In the current study, we presented the crystal structure of recombinant VSP1 at 1.8 Å resolution via the selenomethionine single-wavelength anomalous diffraction (SAD). Specifically, an α-helical cap domain on the top of the α/β core domain is found to be involved in dimerization. In addition, despite of the low sequence similarity between VSP1 and other HAD enzymes, the core domain of VSP1 containing conserved active site and catalytic machinery displays a classic haloacid dehalogenase fold. Furthermore, we found that VSP1 is distinguished from bacterial class C acid phosphatase P4 by several structural features. To our knowledge, this is the first study to reveal the crystal structure of plant vegetative storage proteins.

## Introduction

Vegetative storage proteins (VSPs) are important source of mobilized nutrients for developing plant organs that accumulate in plant vegetative tissues. These proteins are thought to function as temporary storage reserves because of their abundance and patterns of accumulation and degradation. The soybean VSP*α* and VSP*β* are the most characterized VSPs [1], [2]. VSP1 and VSP2 in *Arabidopsis thaliana* share 86% amino acid identity and similar expression patterns to soybean VSPs, and have cross-reaction with the antibodies against soybean VSPs [3].

Apart from their suggested roles in storage, several lines of evidences indicate that *Arabidopsis* VSPs are able to participate in plant defense. For example, they accumulate in response to herbivore damage [4]. In addition, the *vsp1* gene is induced by jasmonate, a plant hormone involved in plant development and defense response [5]. Moreover, study using several *Arabidopsis* mutants indicated that the accumulation of *Arabidopsis* VSPs is involved in the resistance to insect attacks and pathogens [6]. Furthermore, the recombinant *Arabidopsis* VSP2 was found to increase the mortality of insects and delay the development of insects using feeding assay [7]. Finally, *Arabidopsis* VSP1 was found to participate in flower development by interaction with a leucine-rich repeat protein (FLOR1) and the AGMOUS transcription factor, which is required for the stamen and carpel determination of flowers [8].

Based on protein sequence motif analysis, *Arabidopsis* VSP1 and VSP2 are classified as acid phosphatases of the haloacid dehalogenase (HAD) superfamily [9], [10]. Despite a lack of overall sequence similarity (12–22% of identity), *Arabidopsis* VSPs and bacterial class B and C acid phosphatases share a conserved feature motif, called “DDDD” phosphohydrolase due to the presence of four invariant aspartate residues [10]. In this protein family, AphA [11] and P4 [12] are known as the prototypes of class B and class C bacterial nonspecific acid phosphatases, respectively. VSP1 is a representative of the DDDD superfamily in plant.

Early studies indicate that the enzymes of HAD superfamily have a core domain and a “cap” domain (or set of inserts) [13]. The core domain contains the conserved active site and is thus responsible for catalytic activity whereas the cap domain is responsible for the diversification of substrate recognition. Notably, all phosphatase members of the HAD superfamily share a two-step mechanism. The first step is the nucleophilic attack of an aspartate on the phosphate of the phosphoryl group under general acidic catalysis and the second step is the hydrolysis of the aspartyl-phosphate intermediate [13], [14].

In this study, we characterized the acid phosphatase activities of VSP1 and VSP2 and reported a 1.8 Å crystal structure of VSP1. This plant VSP structure not only provides more information for HAD superfamily but also help in exploring potential roles of VSPs in plant defense and development.

## Methods

### Protein expression and purification

The recombinant VSP1 was expressed in *E.coli* and purified as described previously [15]. The production method of VSP2 was the same as that of VSP1 except that the VSP2 protein was eluted in a buffer containing 50 m*M* Tris-HCl (pH 7.5) and 500 m*M* NaCl.

### Activity assay

The phosphatase activity was determined with *p*-nitrophenyl phosphate (*p*NPP) in 50 m*M* sodium acetate buffer (pH 4.5). Two hundred microliters of the reaction mixture containing 10 m*M p*NPP, 10 m*M* MgCl_2_ and the purified protein (0.017 µ*M*) were incubated at 310 K for 30 min and quenched by addition of 1.8 ml of 1.0 M Na_2_CO_3_. The released *p*-nitrophenol was detected by measuring the increase in absorbance at 410 nm and the quantity was calculated according to a standard curve. One unit of the phosphatase activity was defined as the amount of enzyme required to convert 1 µmol of substrate to its product per minute at 310 K.

The steady-state kinetic parameters (*K*m and *K*cat) were determined from initial reaction velocities measured at varying *p*NPP concentrations (ranging from 1 m*M* to 50 m*M*). The initial velocities were measured for reaction mixtures containing 0.035 µ*M* VSP1, 1 or 10 m*M* metal ions in 50 m*M* sodium acetate buffer (pH 4.5) at 310 K. The reaction was quenched by addition of 1.0 *M* Na_2_CO_3_ after 1.5 min, 3.0 min, 4.5 min and 6.0 min. The initial rate was estimated by fitting the data from the four time points to a line. Data at each concentration were collected in triplicate and were fit to the Michaelis-Menten equation (*V*_0_ = *V*max[S]/*K*m+[S], where *V*_0_ is the initial velocity, *V*max is the maximal velocity, *K*m is the Michaelis constant for the substrate and [S] is the concentration of the substrate.) using the nonlinear least-squares-fitting analysis of GraphPad Prism 5 software. The pH of the assay system was measured and found constant before and after adding metal ions.

Inhibition of the enzymatic activity was tested by adding each inhibitor (vanadate, molybdate, inorganic phosphate) to the reaction mixture at concentrations (0.1 m*M*–5.0 m*M* ) and assessed for the effects on the enzymatic activity of VSP1 (0.017 µ*M*), using *p*NPP as the substrate. The IC_50_ (calculated concentration of inhibitor expected to inhibit enzymatic activity by 50%) were obtained through analysis of reduced enzymatic activities using GraphPad Prism 5 software. Each inhibitor was dissolved in distilled H_2_O and none of them will change the system's pH by pH measurement prior to the inhibition assay.

The substrate specificity of VSP1 and VSP2 was determined by measuring the release of inorganic phosphate from different substrates (Table 1) according to the literature [16]. The 1 ml standard assay mixture contained 50 mM sodium acetate (pH 4.5), 10 m*M* MgCl_2_, 2.3 µg (0.08 nmol) purified enzyme and each substrate at 1 m*M* concentration. After incubation for 15 min at 310 K, the assay was stopped by the addition of 1 ml of color reagent [0.12% Malachite Green in 3 *M* H_2_SO_4_, 7.5% ammonium molybdate (10: 2.5, by vol.)]. After color development for 20 min at 310 K, the absorbance at 630 nm was measured with a spectrophotometer for quantification of Pi. A standard curve was generated with concentrations ranging from 0 to 70 nmol of Pi using a 100 µ*M* stock of NaH_2_PO_4_ in 50 m*M* sodium acetate (pH 4.5).

**Table 1:** Relative activities of VSP1 and VSP2 toward different substrates.

Substrate	Hydrolysis activity %	
	VSP1	VSP2
*p*NPP	100	100
MUP	54.8	8.14
phenyl phosphate	5.24	2.93
5′-AMP	ND	0.34
5′-UMP	ND	0.4
5′-GMP	2.84	0.53
5′-CMP	0.13	ND
Glucose 6-phosphate	1.02	0.32
Glucose 1-phosphate	ND	0.12
Glycerol phosphate	ND	ND
Phospho-L-serine	ND	ND
Phosphor-L threonine	2.02	ND
Phosphor-L-tyrosine	0.74	0.81
Phosphor-L-phenylalanine	4.51	1.20
ATP	ND	1.0
ADP	ND	0.736
GTP	ND	0.37
GDP	ND	0.75
NMN	ND	ND
FMN	ND	ND
NADP	ND	ND
cAMP	ND	0.1

ND, not detectable.

Relative activities are expressed as the percentage of the activity with *p*NPP. The results are the average of the values determined in triplicates and the respective standard error is constantly lower than 10%.

### Selenomethionine derivatives preparation and crystallization

Because VSP1 contains no methionine in its primary sequence, VSP1-Met-mutant was constructed with three rounds of site-directed mutagenesis reactions in which Lys87, Val118 and Lys232 were mutated to methionines. The plasmid containing mutated VSP1 was transferred into *E.coli* strain B834. The host cells were grown in M9 minimal medium supplemented with ampicillin and selenomethionine. When OD_600_ of the culture reached 0.6–0.8, IPTG was added to a final concentration of 0.8 m*M* to induce the mutated protein to express for 22 hours at 295 K. The purification protocol of selenomethionine-derived VSP1 was the same as that of the native protein. The Se-Met-labeled VSP1 crystals were obtained with the hanging-drop vapor-diffusion method at 277 K within 3–5 weeks. Hanging drops were prepared by mixing 2 ul of protein solution containing 8 mg/ml protein in 20 m*M* Tris-HCl (pH 8.5) and 10 m*M* NaCl with 1 ul of reservoir solution containing 0.1 *M* sodium citrate tribasic dihydrate (pH 5.6), 5% (v/v) 2-propanol and 18% (w/v) polyethylene glycol 4,000. After optimization, crystals for data collection were obtained.

The VSP1-Mg^2+^ crystals were obtained by the sitting-drop vapor-diffusion method at 277 K within 4 weeks. Sitting drops were prepared by mixing 1 ul of protein solution containing 6.5 mg/ml native protein in 20 m*M* Tris-HCl (pH 7.5), and 10 m*M* NaCl with 1 ul of reservoir solution containing 0.1 *M* sodium acetate (pH 5.5), 80 m*M* MgCl_2_, 7% (*ν*/*ν*) 2-propanol and 12% (w/*ν*) polyethylene glycol 4,000. After optimization, crystals for data collection were obtained.

### Data collection, processing and structure determination

A SAD data set at 0.9789 Å with a selenomethionyl VSP1 crystal was collected at 100 K on Beamline BL17U1 of Shanghai Synchrotron Radiation Facility. The dataset was processed with HKL2000 [17]. The crystal belongs to the space group ***C***2. Three expected selenium atoms were located and used for phase determination at 2.8 Å by Phenix [18], in which density modification was employed for initial model building. A native data set at 0.9789 Å was also collected at 100 K on Beamline BL17U1 of Shanghai Synchrotron Radiation Facility, and processed to 1.8 Å. The initial model was refined with these native data by Coot [19], CNS [20], [21] and Refmac [22] for additional model building and adjustment. In the final model, the electron density of residues Val16-Glu48 was invisible, and therefore, these residues were excluded. The statistics of data reduction and structure refinement are listed in Table 2. The crystal structure of VSP1 has been deposited in the Protein Data Bank under accession number 4FYP. The PDB summary and validation report of this structure is given in the supporting information (Table S1 and S2).

**Table 2:** Statistics of Data Reduction and Structure Refinement.

Data collection statistics	Se-VSP1	Native VSP1
Space group	***C***2	***C***2
Unit cell parameter	***a*** = 128.0, ***b*** = 96.6, ***c*** = 85.5, ***β*** = 90.2°	***a*** = 122.3, ***b*** = 49.2, ***c*** = 84.9, ***β*** = 116.1°
Wavelength (Å)	0.9789	0.9789
Resolution range (Å)^a^	30-2.50(2.59-2.50)	50-1.80(1.86-1.80)
No. of total reflections	151,640	172,556
No. of unique reflections	34,672	41,290
Average redundancy	4.4(4.3)	4.2(4.2)
Completeness (%)	96.8(93.2)	97.2(96.2)
*R*_merge_ b	0.101(0.500)	0.053(0.421)
I/σ	15.4(2.75)	25.6(3.4)
Refinement statistics		
*R*_work_/*R*_free_ (%)^c^	18.1/22.4	
RMSD bond length (Å)^d^	0.011	
RMSD bond angle (°)	1.36	
No. of protein residues	449	
No. of magnesium ions	2	
No. of water molecules	345	
Average temperature factor (Å^2^)		
protein main-chain atoms	31.7	
protein side-chain atoms	35.2	
magnesium ions	26.6	
water molecules	40.6	
Ramachandran plot (%)		
Residues in favored region	96.5	
Residues in allowed region	3.5	
Residues in outlier region	0	

a Data for the highest resolution bin is in parentheses.

b *R*_merge_ = Σ|Ii−Im|/ΣIi, where Ii is the intensity of the measured reflection and Im is the mean intensity of all symmetry-related reflections.

c *R*_work_ = Σ| |Fobs|−|Fcalc| |/Σ|Fobs|, where Fobs and Fcalc are observed and calculated structure factors, respectively. *R*_free_ = Σ_T_| |Fobs|−|Fcalc| |/Σ_T_|Fobs|, where T denotes a test data set of about 5% of the total reflections randomly chosen and set aside prior to refinement.

d RMSD = root-mean-square deviation.

## Results and Discussion

### Acid phosphatase activities of recombinant VSP1 and VSP2

Both recombinant VSP1 and VSP2 exhibited acid phosphatase activity. In addition, we found that divalent metal ions, including Cu^2+^, Mg^2+^, Zn^2+^ and Ni^2+^, were able to enhance the enzymatic activity whereas little activity was observed when no cation was added. These results suggest that divalent metal cation is required for the activities of VSP1 and VSP2. The kinetic parameters of VSP1 were summarized in Table 3.

**Table 3:** Kinetic parameters for VSP1 protein with different divalent metal ions.

Metal ions	*K*m (m*M*)	*K*cat (S^−1^)	*K*cat/*K*m(S^−1^ *M^−^* ^1^)
Cu^2+^ (1 m*M*)	9.431±1.551	25.45±1.755	2698.55
Mg^2+^ (10 m*M*)	14.10 ±2.650	17.75±1.505	1258.87
Zn^2+^ (1 m*M*)	14.82±2.513	12.10±0.8786	816.46
Ni^2+^ (1 m*M*)	16.29±3.538	10.24±1.088	628.61

Data represent the means±SD from three independent assays.

Like many other acid phosphatases, the conventional phosphatase inhibitors, such as vanadate and molybdate, had remarkable inhibitory effects for VSP1 and VSP2. By using *p*NPP (final concentration, 10 m*M*) as a substrate, the IC_50_ was 0.4969±0.0379 m*M* and 0.3022±0.0037 m*M* for molybdate, and 0.5336±0.0069 m*M* and 2.932±0.3864 m*M* for vanadate. By contrast, inorganic phosphate had little effects on the catalytic activity.

The substrate specificity of *Arabidopsis* VSP1 and VSP2 was determined by measuring the release of inorganic phosphate from different substrates. Both VSP1 and VSP2 did not exhibit remarkable activities to any physiological substrates listed in Table 1. The synthetic aryl phosphate substrate *p*NPP was the most readily hydrolyzed substrate. Most other biologically relevant substrates tested were hydrolyzed at less than 5% of the rate of *p*NPP.

### The overall structure of VSP1

**Figure 1:**
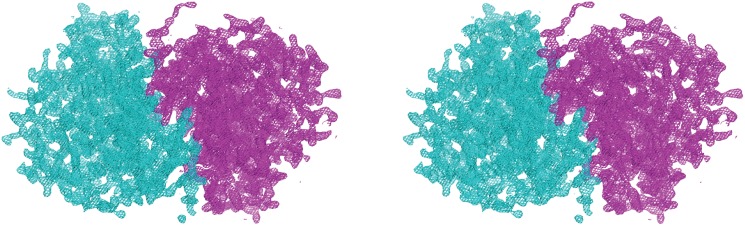
Electron map of VSP1.

**Figure 2:**
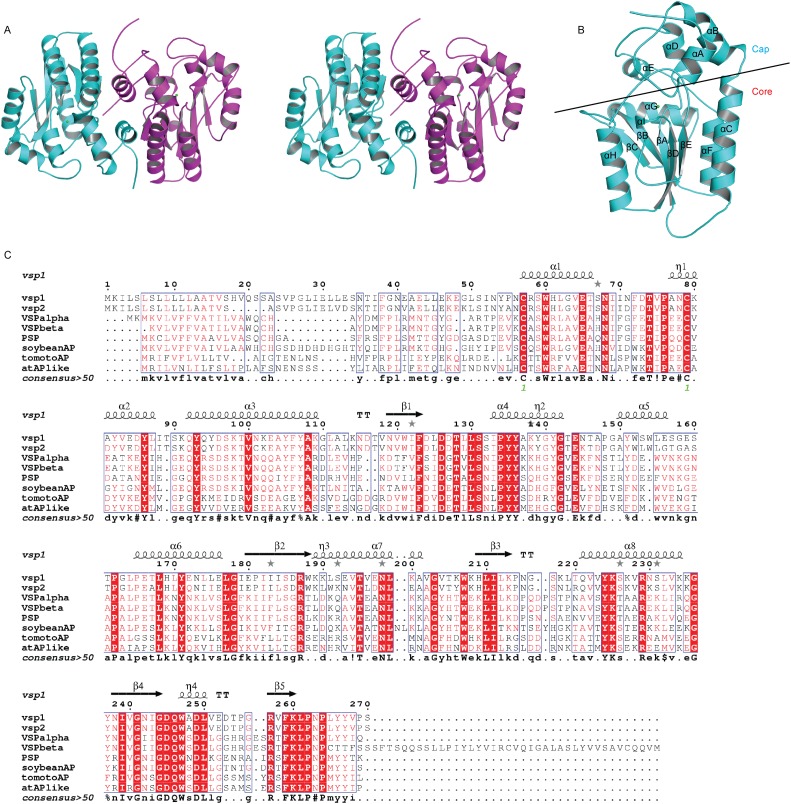
Overall structure of VSP1 and sequence alignment of homologues. (A) Dimeric structure of VSP1. (B) Monomeric structure of VSP1. The core domain and cap-like domain are separated by a line. (C) Sequence aligment of VSP1 with other plant VSPs or plant acid phosphatases. Abbreviation: VSP*α*, soybean VSP*α* (AAA33937); VSP*β*, VSP*β* (AAA34020); PSP, pod storage protein of French Bean (BAA23563); soybeanAP, soybean root nodule phosphatase (CAA11075); tomatoAP, tomato acid phosphatase 1 (CAA39370); atAPlike, Acid phosphatase-like protein of *Arabidopsis thaliana* (AAM14241); VSP1, vegetative storage protein Vsp1 of *Arabidopsis thaliana* (CAC08252).

VSP1 crystallized in space group C2 with two monomers per asymmetric unit (Fig. 1 and 2A). Each monomer had approximate dimensions of 54×31×30 Å^3^. Like most HAD superfamily members, VSP1 structure possessed a two-domain architecture, with a α/*β* core Rossmanoid domain and a smaller α-helical domain (Fig. 2B). Specifically, the core Rossmanoid domain consists of a central five-stranded parallel β-sheets, with an order of βC, βB, βA, βD and βE. In addition, three α-helices (αC, αF and αG) flanked on one side of the β-sheets and two α-helices (αH and αI) were on the other side. Furthermore, the α-helical domain containing four helices, αA, αB, αD and αE, is in a cap-like shape on the top of the α/*β* core domain. The size and location of the cap domain suggests that VSP1 belongs to the classical C1 cap type of the HAD superfamily [9].

A structural homology search using the program DALI [23] conformed that VSP1 is a member of the HAD superfamily. The closest structural homologues are class C bacterial acid phosphatase such as the class C acid phosphatase from *Pasteurella multocida* (PDB code:3pct, Z = 20.2, 21% sequence identity), recombinant *Haemophilus Influenzae* e(P4) acid phosphatase (PDB code: 2hll, Z = 19, 17% sequence identity; PDB code: 3ocu, Z = 18.8, 16% sequence identity), and the class C acid phosphatase from *Bacillus anthracis* (PDB code:2i34, Z = 18.6, 20% sequence identity). Despite the low sequence identity among these proteins, the core domain showed high topological conservation. The well studied lipoprotein P4 from *Haemophilus Influenzae* is the prototype of class C acid phosphatases [12]. The structural comparison between VSP1 and P4 will be described below.

Based on amino acid sequences, there are several VSP1 homologues (39–46% identity in amino acid sequence) in other plants, such as AP-1 from tomato [24], VSP*α* and *β* from soybean [1], root nodule phosphatase from soybean [25], and PSP from French bean [26]. Even in *Arabidopsis thalian*, there are at least eight VSP-like proteins [7]. Most of them have acid phosphatase activities and function as temporary storage reserves or are related to plant defense. Consequently, the structure of VSP1 structure could be the prototype of these plant proteins (Fig. 2C).

### Catalytic sites of VSP1

**Figure 3:**
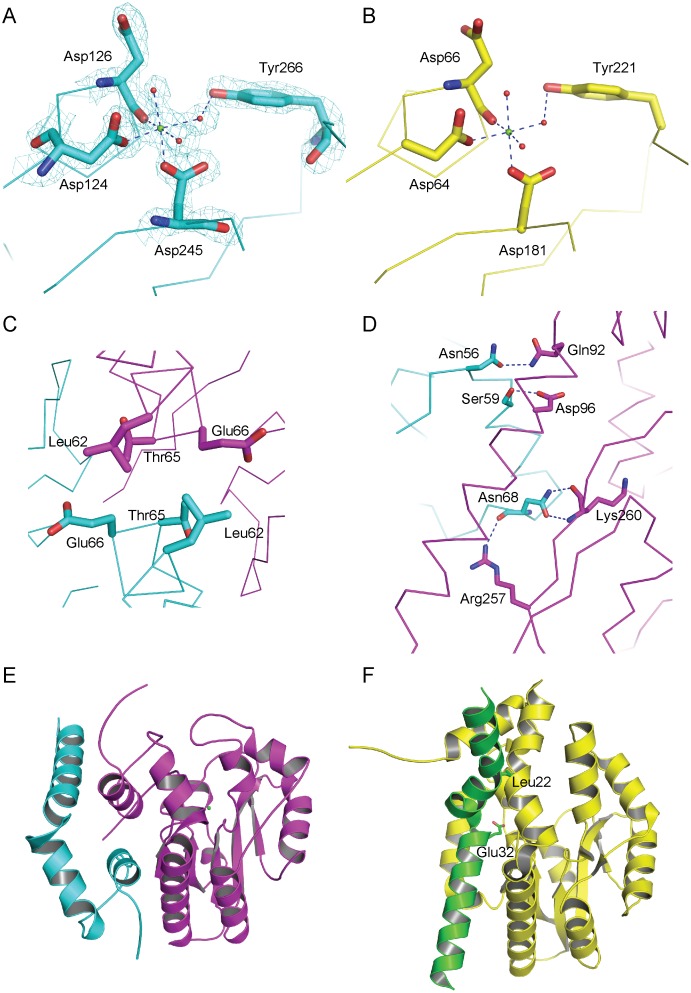
Comparison between VSP1 and P4 in active sites and dimer patterns. (A) Residues in the catalytic site of VSP1. Magnesium ions and water molecules are colored by green and red, respectively. (B) Residues in the catalytic site of P4. Magnesium ion and water molecules are colored by green and red, respectively. (C) Hydrophobic core between two VSP1 monomers. Two VSP1 monomers are colored magenta and cyan, respectively. (D) Hydrogen bonds between two VSP1 monomers. Two VSP1 monomers are colored magenta and cyan, respectively. (E) Interaction pattern of VSP1 dimer. One monomer is coloured magenta, while the N-terminal helices of the other monomer are coloured cyan. (F) Interaction pattern of P4 dimer. One monomer is colored yellow, while the N-terminal helices of the other monomer are colored green. The magenta VSP1 monomer in (E) and the yellow P4 monomer in (F) are aligned.

Consistent with the known members of the HAD superfamily, the active site of VSP1 is located in the cleft between the core and cap domain. Additionally, the core domain contains four catalytic loops that possess the residues responsible for catalyzing and binding to metal ion. Based on the sequence alignment and structure comparison, the four conserved aspartate residues in VSP1 are Asp124, 126, 245 and 249. In addition, a magnesium ion is located at the catalytic center, coordinated by the carbonyl oxygen of Asp126, the carboxyl oxygens of Asp124 and Asp 245, the hydroxyl oxygen of Tyr266 and three water molecules (Fig. 3A). This coordination style is similar to that of P4, one of the closest structural homologues to VSP1. In the P4 structure, the magnesium ion is coordinated by the carboxyl group of Asp64 and Asp181 and the backbone carbonyl oxygen atom of Asp66, plus three water molecules (Fig. 3B).

### Dimeric Structure of VSP1

Recombinant VSP1 is a dimeric enzyme in solution based on the result of gel filtration chromatography, and this was further confirmed by X-ray crystallography (Figure 1 and Figure 2A). According to protein-protein interaction analysis by PISA [27], about 1280 Å^2^ of accessible surface dominated by the interface helix are buried in the monomer interface, corresponding to 11.5% of the total accessible surface. The estimated solvation free energy gain upon formation of the dimer was 212.6 kcal/mol. The dimer interface is built by residues from both core domain and cap domain. The interactions between two monomers in a dimer are attributed to hydrogen bonds and hydrophobic interactions. Residues Asn68 and Lys260*/Arg257*, Asn56 and Gln92*, Ser59 and Asp96* form hydrogen bonds (* indicates residues from neighbouring monomer) respectively, while residues Leu62, Thr66 and Glu65 from both monomers form a hydrophobic core (Figure 3C and 3D). Interestingly, all the residues involved in dimerization in VSP1 also exist in VSP2 based on sequence alignment. It is likely that *Arabidopsis* VSP1and VSP2 can form a heterodimer in the same assembly style of VSP1 homodimer.

P4 is also a dimer but with different dimeric assembly pattern from that of VSP1 (Figure 3E and 3F). In the primary dimer interface of P4, the N-terminal helical elbow of one subunit packs into the groove of the other subunit. This allows the side chains of Glu32 and Leu22 from the helical elbow of one subunit poke through the holes in the dimer interface into the active site of the opposite subunit. These intersubunit contacts most likely stabilize the conformation of catalytic loop and contribute to substrate recognition [12]. In VSP1 structure, the intersubunit surface is located away from the active site and seems not involved in the stability of catalytic site and substrate recognition.

### Structural explanation why VSP1 can't bind NMN

**Figure 4:**
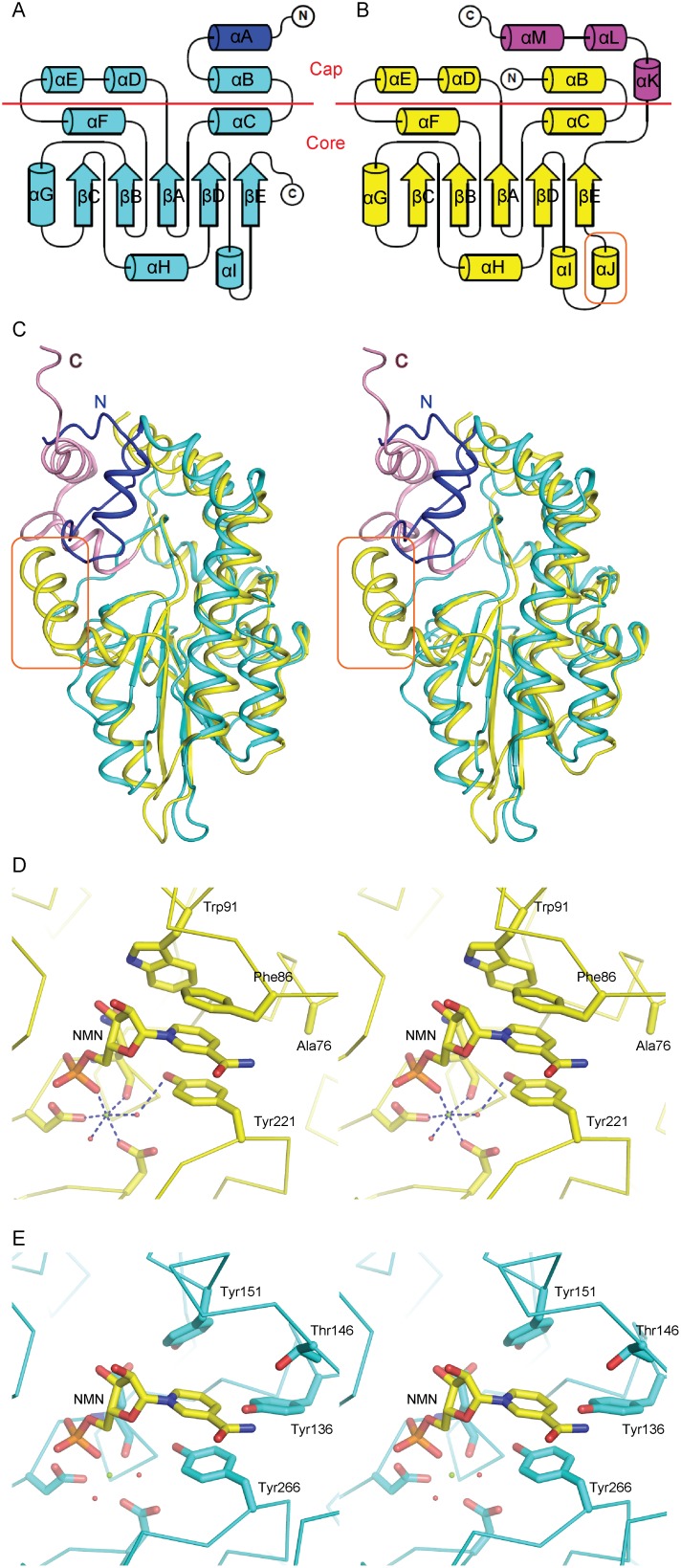
Structural comparison of VSP1 and P4. (A) Topology of VSP1. (B) Topology of P4. (C) Superposition of VSP1 and P4 (stereo view). In (A) (B) (C), an additional α helix in P4 (yellow) is marked by a rectangle. Longer N-terminus in VSP1 (cyan) is colored blue, while longer C-terminus in P4 is colored magenta. Structural elements of P4 in (B) are labeled based on the structural alignment with VSP1. (D) NMN binding with P4. NMN is shown in sticks model. Important residues of P4 interacting with NMN are labeled. (E) Superpose VSP1 to P4 while NMN is modeled at the same site as in P4. Corresponding residues in VSP1 are labeled. NMN is shown in sticks model.

Although the overall structures of VSP1 and P4 are similar, there are two notable differences between them (Figure 4A, 4B and 4C). This may contribute to the different substrate preferences of these two enzymes. Firstly, besides a short α helix between βD and βE strands, as αI in VSP1, one more α helix (named as αJ in figure 4B) exists prior to the βE strand in the P4 structure. Secondly, the N-terminus of VSP1 is longer than that of P4 and contains an additional helix, named as αA. By contrast, the C-terminus of P4 is longer than that of VSP1 with three extra α helices (named as αK, αL and αM in figure 4B). Both the N- and C- terminus of P4 are involved in forming a cap-shape structure, named as “smaller α-helical domain”, whereas the C-terminus of VSP1 does not contribute to the cap domain (Figure 4A, 4B and 4C).

P4 was a nonspecific 5′, 3′-nucleotidase and it dephosphorylates nicotinamide mononucleotide (NMN) to yield nicotinamide riboside (NR) in the NAD^+^ utilization pathway [28], [29]. By comparing the active cleft structure of VSP1 with that of P4-NMN complex, it is noted that the phosphoryl binding site and metal binding site is conserved whereas the upper part of the cleft presumably for the binding to the leaving groups of the substrates is a little different (Figure 3A and 3B, Figure 4D and 4E). Based on the complex structure of P4 with NMN [30], three residues, including Tyr221, Phe86 and Trp91, have hydrophobic interactions with NMN and the base of NMN stacks in parallel between Phe86 and Tyr221, forming an aromatic sandwich (Figure 4D). Furthermore, the span between the aromatic box and phorsphoryl site is optimal for catalysis. After superposing VSP1 to P4, no similar hydrophobic interactions are observed except for Tyr266 (Figure 4E). Nevertheless, Tyr136 in VSP1 may block the entrance of NMN while the entrance in P4 is open because there is an Ala76 in the corresponding site, whose side chain is much smaller than Tyr136 in VSP1 (Figure 4D and 4E). These structural differences between VSP1 and P4 may give an explanation on why VSP1 doesn't exhibit nucleotidase activity. However, the physiological substrates of VSP1 remain unknown in our current study.

## Supporting Information

Table S1 **Summary of PDB entry validation (PDF).**
(PDF)

Table S2 **Validation report of 4FYP (PDF).**
(PDF)
