# Supplementary material for: The Crystal Structure of Arabidopsis VSP1 Reveals the Plant Class C-Like Phosphatase Structure of the DDDD Superfamily of Phosphohydrolases
Source: PLoS One. 2012 Nov 14;7(11):e49421. doi: 10.1371/journal.pone.0049421 (PMC3498132; doi:10.1371/journal.pone.0049421)
Supplement: Table S1 — Summary of PDB entry validation (PDF). (PDF) [file pone.0049421.s001.pdf]

CONFIDENTIAL VALIDATION REPORT

wwPDB Validation Report

PDB ID: 4FYP
RCSB ID: RCSB073518
TITLE: Crystal Structure of Plant Vegetative Storage Protein
AUTHOR(S): Y.Chen, J.Wei, M.Wang, W.Gong, M.Zhang

Summary of PDB Entry Validation

The results of the validation of this PDB entry are shown below. No major issues were raised during data processing.

Geometry Validation

1. **Atomic Clashes**

No issues found.

2. **Peptide Linkage**

No issues found.

3. **Covalent Geometry**

No issues found.

4. **Chirality Error**

No issues found.

CONFIDENTIAL VALIDATION REPORT

Individual Residue Outliers on Real Space R-value

No issues found.

Sequence Validation

The reported biological sequence shows no discrepancy with UniProt sequence (code O49195).

The reported biological sequence and the sequence given in the coordinates show no discrepancy.

Biological Assembly

The biological assembly predicted by PISA is a dimer. This agrees with author's annotation.

Ligand Chemistry

Ligand chemistry has been checked against the Chemical Component Dictionary. The following is a summary.

CONFIDENTIAL VALIDATION REPORT

Identifier: MG
Name: MAGNESIUM ION
Formula: Mg

Type	Program	Version	Descriptor
SMILES	ACDLabs	10.04	[Mg+2]
SMILES CANONICAL	CACTVS	3.341	[Mg++]
SMILES	CACTVS	3.341	[Mg++]
SMILES CANONICAL	OpenEye OEToolkits	1.5.0	[Mg+2]
SMILES	OpenEye OEToolkits	1.5.0	[Mg+2]
InChI	InChI	1.03	InChI=1S/Mg/q+2
InChIKey	InChI	1.03	JLVVSXFLKOJNIY-UHFFFAOYSA-N

CONFIDENTIAL VALIDATION REPORT

Summary of Structure Factor Validation

Structure quality	
Average Real space R-factor (Calculated by SFCHECK, V7.02.4)	0.0836
Average Real space R-factor (Calculated by MAPMAN, V7.8.5)	0.1183
Average Real-space correlation coefficient (Calculated by SFCHECK, V7.02.4)	0.9811
Average Real-space correlation coefficient (Calculated by MAPMAN, V7.8.5)	0.9342
Average Occupancy-weighted avg temperature factor	34.07

Resolution	
High Resolution (Author reported)	1.80
High Resolution (Calculated by SFCHECK, V7.02.4)	1.79
High Resolution (Calculated by REFMAC, V5.5.0109)	1.796
Low Resolution (Author reported)	30.46
Low Resolution (Calculated by SFCHECK, V7.02.4)	30.46
Low Resolution (Calculated by REFMAC, V5.5.0109)	30.463

Crystal data	
Space group	C 1 2 1
Total number of reflections	39150
Number of reflections used	37064
Completeness of data	96.7

R-factors	
R-factor (Author reported)	0.181
R-factor (Calculated by SFCHECK, V7.02.4)	0.206
R-factor (Calculated by REFMAC, V5.5.0109)	0.1857
Free R-factor (Author reported)	0.224
Free R-factor (Calculated by SFCHECK, V7.02.4)	0.249
Free R-factor (Calculated by REFMAC, V5.5.0109)	0.2276

Wilson statistics (PHENIX, V1.6-289)	
Wilson B-factor	27.44
Wilson Scale	0.78

Padilla-Yeates statistics for twin detection (PHENIX, V1.6-289)	
Padilla-Yeates <|L|>	0.495
Padilla-Yeates <L*L>	0.329

CONFIDENTIAL VALIDATION REPORT
