# Supplementary material for: The Crystal Structure of Arabidopsis VSP1 Reveals the Plant Class C-Like Phosphatase Structure of the DDDD Superfamily of Phosphohydrolases
Source: PLoS One. 2012 Nov 14;7(11):e49421. doi: 10.1371/journal.pone.0049421 (PMC3498132; doi:10.1371/journal.pone.0049421)
Supplement: Table S2 — Validation report of 4FYP (PDF). (PDF) [file pone.0049421.s002.pdf]

PDB ID : 4FYP
RCSB ID : RCSB073518
TITLE   : Crystal Structure of Plant Vegetative Storage Protein
AUTHORS : Y.Chen, J.Wei, M.Wang, W.Gong, M.Zhang

The following geometrical and stereochemical features have been calculated for your structure.

CLOSE CONTACTS

==> Close contacts in same asymmetric unit. Distances smaller than 2.2 Angstroms are considered as close contacts.

Chain	Atom	Res	Seq		Chain	Atom	Res	Seq	Symm_Code	Distance
B	O	HOH	454	-	B	O	HOH	550	(1, 5, 5, 5)	Dist = 2.18

==> Close contacts based on crystal symmetry. Distances smaller than 2.2 Angstroms are considered as close contacts.

none

BOND DISTANCES AND ANGLES

Bond and angle checks are performed by first computing the average rms error for all bonds and angles relative to standard values for nucleotide units [L. Clowney et al., Geometric Parameters in Nucleic Acids: Nitrogenous Bases, J.Am.Chem.Soc. 1996, 118, 509-518; A. Gelbin et al., Geometric Parameters in Nucleic Acids: Sugar and Phosphate Constituents, J.Am.Chem.Soc. 1996, 118, 519-529] and amino acid units [R.A. Engh and R. Huber, Structure quality and target parameters, International Tables for Crystallography, Volume F, 2001, 382-392]. Any bond or angle which deviates from the dictionary values by more than six times this computed rms error is identified as an outlier.

==> Covalent Bond Lengths:

The overall RMS deviation for covalent bonds relative to the standard dictionary is   0.013 Angstroms

All covalent bonds lie within a 6.0*RMSD range about the standard dictionary values.

==> Covalent Angle Values:

The overall RMS deviation for covalent angles relative to the standard dictionary is   1.3 degrees.

The following table contains a list of the covalent bond angles greater than 6 times standard deviation.

Deviation	Residue Name	Chain ID	Sequence Number	AT1 - AT2 - AT3	Bond Angle	Dictionary Value	Standard Deviation
14.9	LEU	A	50	CA - CB - CG	130.2	115.3	2.3

1 out of total 5076 bond angles (0.020%) have greater than 6 times standard deviation.

TORSION ANGLES

The torsion angle distributions have been checked. To view these reports,

please refer to the ADIT Validation Server at http://deposit.pdb.org/validate.

==> The following table contains a list of torsion angles outside the expected Ramachandran regions [GJ. Kleywegt and TA. Jones, PHI/PSI−chology: Ramachandran Revisited, Structure 1996, 4, 1395 − 1400].

Residue	Chain	Sequence	PSI	PHI
ILE	A	88	−71.62	−117.56
LEU	A	125	−72.37	−108.49
THR	A	205	−47.37	−135.56
GLU	A	252	39.80	−92.59
ILE	B	88	−72.31	−119.88
LYS	B	91	12.75	59.27
LEU	B	125	−71.60	−104.96
THR	B	128	−57.17	−124.49
GLU	B	252	49.70	−85.50
SER	B	270	−163.49	−113.14

CHIRALITY

The chirality has been checked. O1P, O2P, and hydrogen atoms which do not follow the convention defined in the IUBMB (Liebecq, C. Compendium of Biochemical Nomenclature and Related Documents, 2nd ed.; Portland Press: London and Chapel Hill, 1992) and IUPAC nomenclature (J.L. Markley, A. Bax, Y. Arata, C.W. Hilbers, R. Kaptein, B.D. Sykes, P.E. Wright and K. Wuthrich, Recommendations for the Presentation of NMR Structures of Proteins and Nucleic Acids, Pure & Appl. Chem., Vol. 70, pp. 117−142, 1998) have been standardized. Any other stereochemical violations are listed below.

none

SOLVENT

The following solvent molecules are further than 3.5 Angstroms away from macromolecule atoms in the asymmetric unit that are available for hydrogen bonding. Solvent molecules in extended hydration shells separated by 3.5 Angstroms or less are not listed.

```
HETATM 3761  O   HOH A 491      33.688   0.346  15.633  1.00 51.54      DIST =   3.55 A
HETATM 3944  O   HOH B 499     -22.848 -20.622  26.159  1.00 38.82      DIST =   3.56 A
HETATM 3950  O   HOH B 505     -16.622 -38.906  33.899  1.00 45.51      DIST =   4.80 A
HETATM 3965  O   HOH B 520       1.820 -33.151  31.289  1.00 52.75      DIST =   5.60 A
HETATM 3985  O   HOH B 540       1.221  -2.367  32.286  1.00 60.68      DIST =   3.81 A
```

We have replaced the coordinates for solvent molecules which could be translated back into the asymmetric unit. Please review all solvent molecules in your file and contact us if you have any serious objections.

MISSING RESIDUES

==> The following residues are missing:
(Note: The SEQ number starts from 1 for each chain according to SEQRES sequence record.)

```
RES MOD#C SEQ

VAL(    A  16 )
SER(    A  17 )
HIS(    A  18 )
VAL(    A  19 )
GLN(    A  20 )
SER(    A  21 )
SER(    A  22 )
ALA(    A  23 )
SER(    A  24 )
VAL(    A  25 )
PRO(    A  26 )
GLY(    A  27 )
```

```
  LEU(    A  28 )
  ILE(    A  29 )
  GLU(    A  30 )
  LEU(    A  31 )
  LEU(    A  32 )
  GLU(    A  33 )
  SER(    A  34 )
  ASN(    A  35 )
  THR(    A  36 )
  ILE(    A  37 )
  PHE(    A  38 )
  GLY(    A  39 )
  ASN(    A  40 )
  GLU(    A  41 )
  ALA(    A  42 )
  GLU(    A  43 )
  LEU(    A  44 )
  LEU(    A  45 )
  GLU(    A  46 )
  LYS(    A  47 )
  GLU(    A  48 )
  HIS(    A 277 )
  HIS(    A 278 )
  VAL(    B  16 )
  SER(    B  17 )
  HIS(    B  18 )
  VAL(    B  19 )
  GLN(    B  20 )
  SER(    B  21 )
  SER(    B  22 )
  ALA(    B  23 )
  SER(    B  24 )
  VAL(    B  25 )
  PRO(    B  26 )
  GLY(    B  27 )
  LEU(    B  28 )
  ILE(    B  29 )
  GLU(    B  30 )
  LEU(    B  31 )
  LEU(    B  32 )
  GLU(    B  33 )
  SER(    B  34 )
  ASN(    B  35 )
  THR(    B  36 )
  ILE(    B  37 )
  PHE(    B  38 )
  GLY(    B  39 )
  ASN(    B  40 )
  GLU(    B  41 )
  ALA(    B  42 )
  GLU(    B  43 )
  LEU(    B  44 )
  LEU(    B  45 )
  GLU(    B  46 )
  LYS(    B  47 )
  GLU(    B  48 )
  GLY(    B  49 )
  LEU(    B  50 )
  GLU(    B 272 )
  HIS(    B 273 )
  HIS(    B 274 )
  HIS(    B 275 )
  HIS(    B 276 )
  HIS(    B 277 )
  HIS(    B 278 )


PDB Chain_ID: A

        1                                                         15
SEQRES: VAL SER HIS VAL GLN SER SER ALA SER VAL PRO GLY LEU ILE GLU
COORDS:   ?   ?   ?   ?   ?   ?   ?   ?   ?   ?   ?   ?   ?   ?   ?


        16                                                        30
```

```
SEQRES: LEU LEU GLU SER ASN THR ILE PHE GLY ASN GLU ALA GLU LEU LEU
COORDS:   ?   ?   ?   ?   ?   ?   ?   ?   ?   ?   ?   ?   ?   ?   ?


        31                                                       45
SEQRES: GLU LYS GLU GLY LEU SER ILE ASN TYR PRO ASN CYS ARG SER TRP
COORDS:   ?   ?   ? GLY LEU SER ILE ASN TYR PRO ASN CYS ARG SER TRP
                    49                                           60

        46                                                       60
SEQRES: HIS LEU GLY VAL GLU THR SER ASN ILE ILE ASN PHE ASP THR VAL
COORDS: HIS LEU GLY VAL GLU THR SER ASN ILE ILE ASN PHE ASP THR VAL
        61                                                       75

        61                                                       75
SEQRES: PRO ALA ASN CYS LYS ALA TYR VAL GLU ASP TYR LEU ILE THR SER
COORDS: PRO ALA ASN CYS LYS ALA TYR VAL GLU ASP TYR LEU ILE THR SER
        76                                                       90

        76                                                       90
SEQRES: LYS GLN TYR GLN TYR ASP SER LYS THR VAL ASN LYS GLU ALA TYR
COORDS: LYS GLN TYR GLN TYR ASP SER LYS THR VAL ASN LYS GLU ALA TYR
        91                                                      105

        91                                                      105
SEQRES: PHE TYR ALA LYS GLY LEU ALA LEU LYS ASN ASP THR VAL ASN VAL
COORDS: PHE TYR ALA LYS GLY LEU ALA LEU LYS ASN ASP THR VAL ASN VAL
        106                                                     120

        106                                                     120
SEQRES: TRP ILE PHE ASP LEU ASP ASP THR LEU LEU SER SER ILE PRO TYR
COORDS: TRP ILE PHE ASP LEU ASP ASP THR LEU LEU SER SER ILE PRO TYR
        121                                                     135

        121                                                     135
SEQRES: TYR ALA LYS TYR GLY TYR GLY THR GLU ASN THR ALA PRO GLY ALA
COORDS: TYR ALA LYS TYR GLY TYR GLY THR GLU ASN THR ALA PRO GLY ALA
        136                                                     150

        136                                                     150
SEQRES: TYR TRP SER TRP LEU GLU SER GLY GLU SER THR PRO GLY LEU PRO
COORDS: TYR TRP SER TRP LEU GLU SER GLY GLU SER THR PRO GLY LEU PRO
        151                                                     165

        151                                                     165
SEQRES: GLU THR LEU HIS LEU TYR GLU ASN LEU LEU GLU LEU GLY ILE GLU
COORDS: GLU THR LEU HIS LEU TYR GLU ASN LEU LEU GLU LEU GLY ILE GLU
        166                                                     180

        166                                                     180
SEQRES: PRO ILE ILE ILE SER ASP ARG TRP LYS LYS LEU SER GLU VAL THR
COORDS: PRO ILE ILE ILE SER ASP ARG TRP LYS LYS LEU SER GLU VAL THR
        181                                                     195

        181                                                     195
SEQRES: VAL GLU ASN LEU LYS ALA VAL GLY VAL THR LYS TRP LYS HIS LEU
COORDS: VAL GLU ASN LEU LYS ALA VAL GLY VAL THR LYS TRP LYS HIS LEU
        196                                                     210

        196                                                     210
SEQRES: ILE LEU LYS PRO ASN GLY SER LYS LEU THR GLN VAL VAL TYR LYS
COORDS: ILE LEU LYS PRO ASN GLY SER LYS LEU THR GLN VAL VAL TYR LYS
        211                                                     225

        211                                                     225
SEQRES: SER LYS VAL ARG ASN SER LEU VAL LYS LYS GLY TYR ASN ILE VAL
COORDS: SER LYS VAL ARG ASN SER LEU VAL LYS LYS GLY TYR ASN ILE VAL
        226                                                     240

        226                                                     240
SEQRES: GLY ASN ILE GLY ASP GLN TRP ALA ASP LEU VAL GLU ASP THR PRO
COORDS: GLY ASN ILE GLY ASP GLN TRP ALA ASP LEU VAL GLU ASP THR PRO
        241                                                     255

        241                                                     255
```

```
SEQRES: GLY ARG VAL PHE LYS LEU PRO ASN PRO LEU TYR TYR VAL PRO SER
COORDS: GLY ARG VAL PHE LYS LEU PRO ASN PRO LEU TYR TYR VAL PRO SER
        256                                                     270

        256                         263
SEQRES: LEU GLU HIS HIS HIS HIS HIS HIS
COORDS: LEU GLU HIS HIS HIS HIS   ?   ?
        271                 276


PDB Chain_ID: B

        1                                                        15
SEQRES: VAL SER HIS VAL GLN SER SER ALA SER VAL PRO GLY LEU ILE GLU
COORDS:   ?   ?   ?   ?   ?   ?   ?   ?   ?   ?   ?   ?   ?   ?   ?


        16                                                       30
SEQRES: LEU LEU GLU SER ASN THR ILE PHE GLY ASN GLU ALA GLU LEU LEU
COORDS:   ?   ?   ?   ?   ?   ?   ?   ?   ?   ?   ?   ?   ?   ?   ?


        31                                                       45
SEQRES: GLU LYS GLU GLY LEU SER ILE ASN TYR PRO ASN CYS ARG SER TRP
COORDS:   ?   ?   ?   ?   ? SER ILE ASN TYR PRO ASN CYS ARG SER TRP
                            51                                   60

        46                                                       60
SEQRES: HIS LEU GLY VAL GLU THR SER ASN ILE ILE ASN PHE ASP THR VAL
COORDS: HIS LEU GLY VAL GLU THR SER ASN ILE ILE ASN PHE ASP THR VAL
        61                                                       75

        61                                                       75
SEQRES: PRO ALA ASN CYS LYS ALA TYR VAL GLU ASP TYR LEU ILE THR SER
COORDS: PRO ALA ASN CYS LYS ALA TYR VAL GLU ASP TYR LEU ILE THR SER
        76                                                       90

        76                                                       90
SEQRES: LYS GLN TYR GLN TYR ASP SER LYS THR VAL ASN LYS GLU ALA TYR
COORDS: LYS GLN TYR GLN TYR ASP SER LYS THR VAL ASN LYS GLU ALA TYR
        91                                                      105

        91                                                      105
SEQRES: PHE TYR ALA LYS GLY LEU ALA LEU LYS ASN ASP THR VAL ASN VAL
COORDS: PHE TYR ALA LYS GLY LEU ALA LEU LYS ASN ASP THR VAL ASN VAL
        106                                                     120

        106                                                     120
SEQRES: TRP ILE PHE ASP LEU ASP ASP THR LEU LEU SER SER ILE PRO TYR
COORDS: TRP ILE PHE ASP LEU ASP ASP THR LEU LEU SER SER ILE PRO TYR
        121                                                     135

        121                                                     135
SEQRES: TYR ALA LYS TYR GLY TYR GLY THR GLU ASN THR ALA PRO GLY ALA
COORDS: TYR ALA LYS TYR GLY TYR GLY THR GLU ASN THR ALA PRO GLY ALA
        136                                                     150

        136                                                     150
SEQRES: TYR TRP SER TRP LEU GLU SER GLY GLU SER THR PRO GLY LEU PRO
COORDS: TYR TRP SER TRP LEU GLU SER GLY GLU SER THR PRO GLY LEU PRO
        151                                                     165

        151                                                     165
SEQRES: GLU THR LEU HIS LEU TYR GLU ASN LEU LEU GLU LEU GLY ILE GLU
COORDS: GLU THR LEU HIS LEU TYR GLU ASN LEU LEU GLU LEU GLY ILE GLU
        166                                                     180

        166                                                     180
SEQRES: PRO ILE ILE ILE SER ASP ARG TRP LYS LYS LEU SER GLU VAL THR
COORDS: PRO ILE ILE ILE SER ASP ARG TRP LYS LYS LEU SER GLU VAL THR
        181                                                     195

        181                                                     195
SEQRES: VAL GLU ASN LEU LYS ALA VAL GLY VAL THR LYS TRP LYS HIS LEU
COORDS: VAL GLU ASN LEU LYS ALA VAL GLY VAL THR LYS TRP LYS HIS LEU
```

```
        196                                                         210

        196                                                         210
SEQRES: ILE LEU LYS PRO ASN GLY SER LYS LEU THR GLN VAL VAL TYR LYS
COORDS: ILE LEU LYS PRO ASN GLY SER LYS LEU THR GLN VAL VAL TYR LYS
        211                                                         225

        211                                                         225
SEQRES: SER LYS VAL ARG ASN SER LEU VAL LYS LYS GLY TYR ASN ILE VAL
COORDS: SER LYS VAL ARG ASN SER LEU VAL LYS LYS GLY TYR ASN ILE VAL
        226                                                         240

        226                                                         240
SEQRES: GLY ASN ILE GLY ASP GLN TRP ALA ASP LEU VAL GLU ASP THR PRO
COORDS: GLY ASN ILE GLY ASP GLN TRP ALA ASP LEU VAL GLU ASP THR PRO
        241                                                         255

        241                                                         255
SEQRES: GLY ARG VAL PHE LYS LEU PRO ASN PRO LEU TYR TYR VAL PRO SER
COORDS: GLY ARG VAL PHE LYS LEU PRO ASN PRO LEU TYR TYR VAL PRO SER
        256                                                         270

        256                         263
SEQRES: LEU GLU HIS HIS HIS HIS HIS HIS
COORDS: LEU   ?   ?   ?   ?   ?   ?   ?
        271
```
